# Acute Pancreatitis in Children: The Clinical Profile at a Tertiary Hospital

**DOI:** 10.7759/cureus.14871

**Published:** 2021-05-06

**Authors:** Saeed Al Hindi, Zahra Khalaf, Khaled Nazzal, Osama Nazzal, Alya Ahmed, Lama Alshaibani

**Affiliations:** 1 Department of Pediatric Surgery, Salmaniya Medical Complex, Manama, BHR; 2 Department of Surgery, Ibn Al-Nafees Hospital, Manama, BHR; 3 Medicine, College of Medicine and Medical Sciences, Arabian Gulf University, Manama, BHR; 4 Department of Internal Medicine, King Hamad University Hospital, Manama, BHR

**Keywords:** cholelithiasis, complications, etiology, parenteral, sickle cell disease, acute pancreatitis, clinical profile, pancreatitis, pediatric, laboratory markers

## Abstract

Objectives

The clinical course and progression of acute pancreatitis are poorly understood to date, necessitating more studies of clinical profiles during the disease. Moreover, understanding the etiologies and clinical presentations of acute pancreatitis (AP) in children can contribute to early diagnosis and, hence, earlier interventions. Therefore, this article aims to study the clinical profiles of children with acute pancreatitis (AP) in relation to complications and other variables.

Study design

We retrospectively studied 56 patients who presented with AP to the pediatric department in Salmaniya Medical Complex between January 2006 and December 2017. Cases of chronic pancreatitis and ages above 12 years were excluded. The data concerned demographics, etiology, clinical data, hospital course, and outcomes.

Results

The study included 56 patients aged a mean of 8.46 years (male:female - 33:23). The average hospital stay was 7.68 days. Patients received parenteral feeds a mean of 2.77 days. All patients had an ultrasound, nine required CT scans (16.1%), and five MRIs (8.9%). There were 18 local complications (32.1%): pseudocysts (n=3, 5.36%), cholangitis (n=2, 3.6%), and edema (n=13, 23.2%). There were 23 intensive care unit (ICU) admissions (41.1%). No mortalities occurred but there were six recurrences (10.7%). Symptoms of abdominal pain, vomiting, fever, and nausea occurred in 100%, 57.1%, 35.7%, and 23.2% of patients, respectively. Etiologies were 41.1% biliary, 23.2% idiopathic, 19.6% traumatic, and 8.93% drug-induced. Leukocytes were elevated in 20 patients (35.7%), c-reactive protein (CRP) in five (8.93%), serum amylase in 45 (80.4%), and urinary amylase in all 56 patients (100%).

Conclusion

Most pediatric AP cases were attributed to biliary causes followed by trauma. Age was significantly correlated with complication rates (P=0.013). Abdominal pain was a more common symptom than vomiting. Leukocytosis was associated with ICU admissions. There was no significant relation between c-reactive protein, serum amylase, or urinary amylase, and complications or ICU admissions. Pediatric AP was self-limiting and there were no fatalities.

## Introduction

Acute pancreatitis (AP) can be defined by the presence of characteristic abdominal pain, an increase in pancreatic enzymes by a factor of 3, and suggestive imaging findings. According to the ‘Atlanta’ and ‘International Study Group of Paediatric Pancreatitis: In Search of a Cure’ (INSPPIRE) criteria, the diagnosis of AP is made when two of these three features are present [1-2].

There has been an increasing trend in pancreatitis among pediatric populations over the years. This increase has been linked to a greater understanding of etiologies and advancements in diagnostic modalities [3-4]. The causes of AP in children have been classified as anatomic, biliary, infectious, traumatic, toxic, metabolic, associated with systematic illness, familial, metabolic, drug-induced, and idiopathic [5]. Overall, AP in the pediatric age group has a good prognosis and a low mortality rate, yet there is a risk of recurrence in 15% to 35% of patients (North American Society for Pediatric Gastroenterology, Hepatology, and Nutrition (NASPGHAN) [6].

Despite a better understanding of pediatric pancreatic causes over the years, disease progression and course remain poorly understood. Moreover, enhanced understanding of etiologies, how common they are, and associated factors can further improve detection and, hence, outcomes on both a local and international level. In this study, our objective was to study the clinical profile of patients with acute pancreatitis and correlate laboratory markers and other variables with complications.

## Materials and methods

This study was conducted retrospectively, including all patients diagnosed with acute pancreatitis who were admitted to the pediatric department in Salmaniya Medical Complex (a tertiary hospital) between January 2006 and December 2017. Patients aged above 12 years of age and those with chronic pancreatitis were excluded.

We reviewed the patients’ medical records, including data from their history and clinical examination notes and their laboratory and radiological investigations. The data collected included demographics (age and sex), etiology, clinical presentation, diagnosis, hospital course (hospital stay, intensive care unit (ICU) admission, and ‘nil per os’ (NPO) state), laboratory investigations, complications (imaging findings), recurrence rates, and follow-up durations. Patients were allocated to etiologies based on the registered cause of AP in the records. Cases were labeled idiopathic pancreatitis if no etiology was found. Biochemical markers were collected from records from the time of hospital admission. All sickle cell disease patients were admitted to the ICU as soon as they were diagnosed with AP, in compliance with our hospital guidelines, due to their tendencies to quickly deteriorate.

Follow-up was performed for all patients for variable durations ranging from four to 59 months (mean: 39.4 months).

Diagnosis, imaging protocol, and complications

The diagnosis of acute pancreatitis was made according to the Atlanta and INSPPIRE criteria [1]. The criteria include clinical presentations suggestive of AP (epigastric or right upper quadrant abdominal pain with or without radiation to the back), elevated serum amylase and/or lipase by at least three times the upper normal limit, and imaging findings on ultrasound or computed tomography (CT) scans suggestive of AP. A diagnosis was made if two of the three criteria were met.

Abdominal ultrasounds were the imaging modality used. CT and magnetic resonance imaging (MRI) were only used when the ultrasound findings necessitated further evaluation.

Moreover, information about local complications was gathered. According to the Atlanta classification, local complications include: “acute peripancreatic fluid collection, pancreatic pseudocyst, acute necrotic collection, and walled-off necrosis” [1].

Statistical analysis

The Statistical Package of the Social Sciences (SPSS) statistics software, version 27 (IBM Corp, Armonk, NY), was used for statistical analysis. Frequency tables were used to present descriptive statistics. Chi-square tests were used to correlate variables with complications. Variable correlations were deemed statistically significant if the P-value was 0.05 or less. Crosstabulation was used to assess variables analyzed in terms of categories.

Ethical considerations

This study received ethical approval from the Ministry of Health Research Committee.

## Results

This study examined 56 patients presenting to the pediatric department with AP. Among these, there were 33 males (58.9%) and 23 females (41.1%). Sex was not significantly correlated with complications (P=0.36), recurrence (P=0.063), or ICU stay (P=0.98). The patients had a median of eight years of age at the time of diagnosis (interquartile range (IQR): 3 years). Furthermore, age was categorically divided into ranges: six (10.7%) of the patients were aged five years or less, 39 (69.6%) were between six and 10 years, and 11 (19.6%) were between 11 and 12 years. A significant association was found between age and complication rates (P=0.013) (Table 1).

**Table 1:** Patient demographics in relation to complications, recurrence, and ICU admission ICU: intensive care unit

	Frequency N (%)	Complications N (%)	Recurrence N (%)	ICU N (%)
Gender		(P= 0.36)	(P= 0.063)	(P= 0.98)
Males	33 (58.9)	1 (3)	3 (9.1)	13 (39.4)
Females	23 (41.1)	2 (8.7)	3 (13)	10 (43.5)
Age		(P=0.013)	(P=0.77)	(P=0.31)
0-5 years	6 (10.7)	0 (0)	1 (16.7)	0 (0)
6-10 years	39 (69.6)	3 (7.69)	4 (10.3)	18 (46.2)
11-12 years	11 (19.6)	0 (0)	1 (9.1)	5 (45.5)

Regarding clinical presentations, all 56 patients experienced abdominal pain (100%). The second most common symptom was vomiting (n=32, 57.1%), followed by fever (n=20, 35.7%) and nausea (n=13, 23.2%).

The median duration of their hospital stay was five days (range: 4-30 days; IQR: 3 days). The longest duration of hospitalization was for 30 days for a case of drug-induced AP. The patients did not receive oral intake for a median of 2 days (IQR: 2 days).

Laboratory investigations assessed included leukocyte count, C-reactive protein (CRP), serum amylase, and urinary amylase. Leukocyte count was elevated in 20 patients (35.7%), CRP in five (8.93%), serum amylase in 45 (80.4%), and urinary amylase in all cases (100%). Serum amylase values were most commonly within the range of >500 to ≤1000 U/L (n=26, 46.4%), followed by ranges from >110 to ≤500 U/L (n=19, 33.9%). Urinary amylase values most frequently ranged from >500 to ≤1000 U/L (n=31, 55.4%), followed by values over 1000 U/L (n=20, 35.7%).

Elevated leukocyte count was significantly correlated with ICU admissions (P=0.013). None of the laboratory parameters were significantly correlated with complications or ICU admissions (P>0.05) (Table 2).

**Table 2:** Laboratory biomarkers in relation to complications and ICU admission ICU: intensive care unit

Parameter	N (%)	Complications (%)	ICU admission (%)
Leukocyte count (cumm)		(P= 0.37)	(P=0.013)
≤ 11,000	36 (64.3)	1 (2.8)	13 (36.1)
> 11,000	20 (35.7)	2 (10)	10 (50)
C-reactive Protein (CRP) (mg/dl)		(P= 0.4)	(P=0.068)
≤ 5	51 (91.1)	1 (2)	21 (41.2)
> 5	5 (8.93)	2 (40)	2 (40)
Serum amylase (U/L)		(P= 0.12)	(P=0.24)
≤ 110	11 (19.6)	0 (0)	6 (54.5)
>110 - ≤500	19 (33.9)	2 (10.5)	10 (52.6)
>500 - ≤1000	26 (46.4)	1 (3.8)	7 (26.9)
>1000	0	-	-
Urinary amylase (U/L)		(P= 0.8)	(P=0.17)
≤ 140	0	-	-
>140 - ≤500	5 (8.9)	0 (0)	2 (40)
>500 - ≤1000	31 (55.4)	2 (6.5)	14 (45.2)
>1000	20 (35.7)	1 (5)	7 (35)

All patients had an abdominal ultrasound. Only nine patients (16.1%) required a CT scan and five (8.9%) had MRIs.

The etiologies of AP in this study were biliary, traumatic, drug-induced, systematic, disease-associated, cystic fibrosis, pancreatic anomalies, and idiopathic. None of the cases in this study were attributed to viral etiologies. The most common cause was biliary and was observed in 23 cases (41.07%). Among these, 19 were secondary to cholelithiasis from sickle cell disease (33.9%), three were due to a choledochal cyst (15.4%), and one was a result of congenital spherocytosis (1.8%). Two of the cholelithiasis cases also had cholangitis. The second most common cause category was idiopathic, which was the case in 13 children (23.2%). There were 11 cases of AP attributed to trauma (19.6%): three were due to a blunt force to the abdomen, two were post-endoscopic retrograde cholangiopancreatography (ERCP), and the remaining six were associated with falls. Moreover, five cases were drug-induced (8.93%). There were two occurrences of AP associated with systemic diseases (3.57); one of these was in a case of familial hyperlipidemia and the other was in a case of ascariasis. Furthermore, there was one case of AP in a cystic fibrosis patient (1.79%) and one case of AP in a child with a known pancreatic anomaly (1.79%).

Cholelithiasis in sickle cell disease patients was associated with the longest mean ICU stay (4.58 days), followed by trauma and drug association. All 19 sickle cell patients (82.61%) were admitted to the ICU as a precautionary measure due to tendencies to develop more severe forms of all diseases and fast deterioration (Table 3).

**Table 3:** Correlating the etiology of AP with age, sex distribution, and ICU stay AP: acute pancreatitis; ICU: intensive care unit

Etiology	No. of patients (%)	Mean age (years) P= 0.062	Male: female ratio P= 0.11	Mean ICU stay P= 0.55
Biliary	23 (41.07)	7.05	12:11	-
Sickle Cell Disease	19 (33.9)	9.16	10:9	4.58
Choledochal Cyst	3 (5.4)	4	1:2	0
Congenital Spherocytosis	1 (1.8)	8	1:0	0
Idiopathic	13 (23.2)	8	8:5	0
Trauma	11 (19.6)	9.64	9:2	1.82
Fall injury	6 (10.71)	-	-	-
Blunt abdominal trauma	3 (5.36)			
Post-ERCP trauma	2 (3.57)			
Drug-induced	5 (8.9)	8.2	3:2	1.6
Systemic	2 (3.57)	5	0:2	0
Familial Hyperlipidemia	1 (1.8)	-	-	-
Ascariasis	1 (1.8)			
Cystic Fibrosis	1 (1.79)	9	0:1	0
Pancreatic Anomalies	1 (1.79)	4.5	1:1	0
Total	56 (100)	8.46	33:23	2.05

Local complications occurred in three cases (5.3%); all were cases of pseudocysts. There were no patients with acute necrotic collection, pancreatic or peripancreatic necrosis, or walled-off necrosis (Table 4).

**Table 4:** Local complications in relation to recurrence and ICU admission ICU: intensive care unit

Local complications	Frequency N (%)	Recurrence N (%)	ICU N (%) (P= 0.14)
Pseudocysts	3 (5.3)	0 (0)	2 (66.7)
Acute necrotic collection	0 (0)	-	-
Pancreatic and peripancreatic necrosis	0 (0)	-	-
Walled-off necrosis	0 (0)	-	-

A total of 23 patients (41.1%) were admitted to the ICU; 19 of them were sickle cell disease patients who were admitted to the ICU out of precaution due to fast deterioration tendencies. The ICU stay ranged from zero to eight days. Both patients who had cholelithiasis with cholangitis were admitted to the ICU for eight days.

Furthermore, the patients were followed up for a mean of 39.4 months (range: 4 to 59 months). Following recovery, only six cases (10.7%) experienced a recurrence.

## Discussion

AP in children has shown a growing trend worldwide [7-13]. This may be related to increased awareness of AP etiologies and changes in referral and diagnosis practices. Although most cases of AP are self-limiting, 15% to 34% of pediatric cases are severe [7]. The clinical worsening and progression of AP is a poorly understood field, which necessitates a more extensive study of clinical profiles to help develop improved predictive models. Our study analyzed the clinical profile of AP in children. We found that pediatric AP was more common in males and age between six years and 10 years. The most common symptoms were abdominal pain and vomiting. Regarding biochemical markers, the leukocyte count was high in 20 cases (35.7%) and CRP in five cases (8.93%), serum amylase in 45 cases (80.4), and urinary amylase were elevated in all patients. High leukocytes were correlated with ICU admissions, but no correlations were found between other laboratory markers and complications or ICU admissions. Cholelithiasis was the leading cause of AP in children followed by trauma. Local complications occurred in three individuals (5.3%); all were occurrences of pseudocysts. There were no other forms of complications. Only six patients had a recurrence of acute pancreatitis (10.7%), and there were no mortalities.

We found that AP was more prevalent in males (58.9%), with a male to female ratio of 33:22. Similarly, Poddar et al. and Lal et al. found that AP was more common in males [10,14]. Furthermore, most patients were aged between six and 10 years old (69.6%). Our study found a significant correlation between age and complications (P=0.013), which is similar to findings by other studies about the correlation between age and outcomes [10,13].

In line with the spectrum of pediatric AP causes available in the literature, the etiologies in our study were biliary, traumatic, drug-induced, systematic disease-associated, cystic fibrosis, pancreatic anomalies, and idiopathic [8,11,14]. Similar to findings by other studies [13,15], most of our cases of AP were attributed to biliary causes (n=23, 41.07%), consisting mostly of sickle cell disease-associated cholelithiasis (n=19, 33.9%). Unlike other studies, our study demonstrates a high prevalence of sickle cell disease; this can be explained by the higher regional prevalence of sickle cell disease in comparison to other locations [16]. Trauma accounted for 19.6% of the cases, which is within the range reported by studies, 10% to 40% [17]. However, our percentage of drug-induced AP (8.9%) is lower than the percentage reported by other researchers (13%) [18]; this may be linked to an underreporting of drug-induced cases. Furthermore, 23.2% of our cases were labeled idiopathic, which is lower than the range of idiopathic AP cases in the literature, 34% to approximately 50% [10,19]. The cause most strongly associated with an ICU stay was sickle cell disease cholelithiasis, followed by trauma and drug-induced cases. However, the association between sickle cell cholelithiasis and ICU admissions can be attributed to institutional guidelines that mandate admitting sickle cell patients who develop acute pancreatitis to the ICU to receive more intensive care.

For the clinical presentation, in our study, the most prevalent symptoms were abdominal pain (100%) and vomiting (32%); this is in line with the findings of other studies [8,19-20]. The incidences of fever and nausea were less common, occurring in 35.7% and 23.2% of patients, respectively.

For the hospital course, we found that the average hospital stay was 7.68 days (range: 4-30 days), where the longest duration was due to a drug-associated etiology. Pezzilli et al. reported a longer mean hospitalization period of 13 days [19].

Furthermore, current management practices used in pediatric AP are derived from adult guidelines. Evidence on nutrition management practices in AP has moved from urging parenteral nutrition to enteral nutrition. The most recent evidence has linked early enteral nutrition with lower mortality rates due to decreased infection rates and the prevention of gastrointestinal tract bacterial stasis [21-22]. In our study, ‘nil per os’ (NPO0 status ranged from 1-21 days (mean 2.77 days), during which patients received parenteral feeds. This is comparable to other practices where most pediatric patients were initiated on parenteral feeds (44%) as opposed to enteral feeds (3%) despite the urges to start with enteral feeds [4,15].

For the imaging protocol, all patients were investigated by ultrasound while only those who required further investigation had other forms of imaging performed; nine had a CT scan (16.1%) and five had MRIs (8.9%). This compares to trends in other AP studies in which most of their patients had ultrasounds performed and CT scans were only performed when necessary [20].

Local complications occurred in three patients in our study (5.3%), all were in the form of pseudocysts. There were no cases of necrotic collections, pancreatic or peripancreatic necrosis, or walled-off necrosis. A total of 23 patients (41.1%) in our study required ICU care, 19 of which were sickle cell disease patients who were admitted to the ICU for precaution.

Regarding outcomes, the mortality rates associated with pediatric AP are lower than those in adults, ranging from 0% to 11% [8,20]. There were no mortality cases in our study. However, recurrence occurred in 10% of patients (n=6); this is in line with the rates of recurrence for AP in children, which range from 15% to 35% [8,10,23].

We also assessed biochemical markers. The leukocyte count was increased in 20 patients (35.7%), CRP in five (8.93%), serum amylase in 45 (80.4%), and urinary amylase in all cases (100%). Elevated leukocyte counts were significantly correlated to ICU admissions (P=0.013). All other laboratory markers were not significantly correlated with complication rates or ICU stay (P>0.05). In the literature, serum amylase’s sensitivity in diagnosing AP in children ranged from 50% to 85% while lipase had a slightly higher sensitivity [20]. Moreover, Pezzilli et al. found that higher CRP levels were correlated with severe pancreatitis. Pezzilli also assessed other biochemical markers and found severity to be significantly correlated with lower calcium, higher potassium, and a lower hematocrit [19].

A number of limitations can be found in this study. First, we had a small number of patients. Second, as the ultrasound was the main imaging modality used while it has the advantages of avoiding ionizing radiation and being readily available, its disadvantages include that it is operator-dependent and that pancreatic visualization can be obscured by overlying bowel gas or abdominal fat. Moreover, due to the retrospective nature of the study, only biochemical and clinical markers available in the records could be included; hence lipase and systemic inflammatory response syndrome criteria and drug names in cases of drug-induced AP were not included. Furthermore, we documented only local complications, and specific systemic complications that may have occurred in the ICU were not documented due to the retrospective nature of the study. Additionally, cross-comparison between pediatric studies is limited by variations in the age ranges among pediatric departments worldwide.

## Conclusions

Most cases of AP in the children in our study were self-limiting. The majority of cases were due to biliary etiologies rather than trauma, comprised mostly of sickle cell disease-associated cholelithiasis. The most common symptoms were abdominal pain and vomiting. Elevations in leukocyte counts were correlated with ICU admissions. However, other biochemical markers were not significantly linked to complications or ICU admission. There were three cases complicated by pseudocysts. Moreover, age was significantly associated with the presence of complications. There was a 10.7% recurrence rate and no associated mortalities.
